# Surface Analysis of Biodegradable Mg-Alloys after Immersion in Simulated Body Fluid

**DOI:** 10.3390/ma13071740

**Published:** 2020-04-08

**Authors:** Darja Steiner Petrovič, Djordje Mandrino, Božidar Šarler, Jelena Horky, Andrea Ojdanic, Michael J. Zehetbauer, Dmytro Orlov

**Affiliations:** 1 Physics and Chemistry of Materials, and Simulation of Materials and Processes, Institute of Metals and Technology, Lepi pot 11, 1000 Ljubljana, Slovenia; Djordje.Mandrino@imt.si (D.M.); Bozidar.Sarler@imt.si (B.Š.); 2 Department of Fluid Dynamics and Thermodynamics, Faculty of Mechanical Engineering, University of Ljubljana, Aškerčeva 6, 1000 Ljubljana, Slovenia; 3 Center for Health & Bioresources, Biomedical Systems, AIT Austrian Institute of Technology, Viktor Kaplan Straße 2, 2700 Wiener Neustadt, Austria; Jelena.Horky@ait.ac.at; 4 Physics of Nanostructured Materials, Faculty of Physics, University of Vienna, Boltzmanngasse 5, 1090 Vienna, Austria; Andrea.Ojdanic@univie.ac.at (A.O.); Michael.Zehetbauer@univie.ac.at (M.J.Z.); 5 Division of Materials Engineering, Department of Mechanical Engineering, Faculty of Engineering (LTH), Lund University, Ole Römers väg 1, 223 63 Lund, Sweden; Dmytro.Orlov@material.lth.se

**Keywords:** biodegradable Mg alloy, corrosion, X-ray photoelectron spectroscopy, phosphate, apatite, degradation layer

## Abstract

Two binary biodegradable Mg-alloys and one ternary biodegradable Mg-alloy (Mg-0.3Ca, Mg-5Zn and Mg-5Zn-0.3Ca, all in wt%) were investigated. Surface-sensitive X-ray photoelectron spectroscopy analyses (XPS) of the alloy surfaces before and after immersion in simulated body fluid (SBF) were performed. The XPS analysis of the samples before the immersion in SBF revealed that the top layer of the alloy might have a non-homogeneous composition relative to the bulk. Degradation during the SBF immersion testing was monitored by measuring the evolution of H_2_. It was possible to evaluate the thickness of the sample degradation layers after the SBF immersion based on scanning electron microscopy (SEM) of the tilted sample. The thickness was in the order of 10–100 µm. The typical bio-corrosion products of all of the investigated alloys consisted of Mg, Ca, P and O, which suggests the formation of apatite (calcium phosphate hydroxide), magnesium hydrogen phosphate hydrate and magnesium hydroxide. The bioapplicability of the analyzed alloys with regard to surface composition and degradation kinetics is discussed.

## 1. Introduction

Artificial materials considered to be suitable for applications as implants are commonly tested in vivo and by in vitro methods in media that simulate body fluids. These tests are focused on an examination of the physical, chemical and mechanical properties of the materials and thus provide the basic information needed to judge their suitability for clinical use in the human body [1]. Currently, magnesium-based biodegradable alloys are gaining importance as temporary implant materials in orthopedic applications [2,3,4]. Their elastic properties are similar to those of bones, and their in vivo biodegradability through corrosion makes them candidates for different biomedical applications such as biocompatible, osteoconductive, degradable implants for load-bearing and bone repairing [5]. The major drawback of using Mg alloys as implant materials is their too-high corrosion rate in a physiological environment, which can impair their mechanical properties [6,7,8,9]. This is mainly addressed by surface coating and optimizing the alloying of Mg [10,11,12,13].

One of the alloying elements used for Mg is Ca, and both are crucial for the formation of human bone [3,6,14,15,16,17]. Mg and Ca are essential quantity elements and represent two of the four main electrolytes that constitute about 1.89% of an average human body, while the remaining 0.02% is made up of eleven typical trace elements. Zn is an essential trace element and can be found in all body tissues, with 85% of all of the body’s zinc in the bone and muscle [18]. Zn is known to have a positive impact on bone healing and cell reactions [19].

It has been established that Mg-Ca alloys have good biocompatibility due to their degradation in bone [3,16,17]. Mg alloys with Ca or Zn, or ternary Mg-Zn-Ca alloys, also possess highly favorable mechanical properties [5,17,20,21,22].

In this study, two binary (Mg-0.3Ca and Mg-5Zn) alloys and one ternary (Mg-5Zn-0.3Ca) alloy were investigated as-cast and homogenized, after in vitro bio-corrosion immersion tests in simulated body fluid (SBF). The chemical reaction took place during immersion, as observed by hydrogen evolution. Reaction products in the form of surface-degradation layers were formed on all of the alloys. These degradation layers were analyzed in detail.

## 2. Experimental

### 2.1. Materials and Sample Preparation

Three Mg alloys, two binary (Mg-0.3Ca and Mg-5Zn, i.e., X03 and Z5, respectively) and one ternary (Mg-5Zn-0.3Ca, i.e., ZX50), with compositions as given in Table 1, were processed as follows: the alloys were cast at the LKR Leichtmetallkompetenzzentrum Ranshofen, Austria, a subsidiary of the AIT Austrian Institute of Technology. From the cast billets with diameters of 50 mm, samples were prepared as disks of magnesium alloys with diameter ϕ = 10 mm and height h = 0.7 mm. They were solid-solution treated at 450 °C for 24 h in the case of the Mg-Ca and Mg-Zn-Ca, and at 350 °C for 12 h in the case of the Mg-Zn. Before immersion into the SBF, with the composition given in Table 2 (i.e., SBF27 from [1]), the samples were polished down to 1200-grit SiC paper and cleaned with ethanol to minimize the oxidation layer and to obtain a defined surface condition before testing.

All of the corrosion experiments were conducted at body temperature, and a Tris/HCl buffer was used to adjust the pH of the SBF to an initial value of approximately 7.4. Two samples were always immersed together in 250 mL of SBF. The SBF was replaced with a fresh batch every 7 days, and the whole test was conducted for 21 days.

### 2.2. Monitoring the Corrosion Reaction in SBF

As a measure of the reaction progress of the alloy in the SBF, the evolving hydrogen gas was collected in a glass cylinder, and the amount was recorded every 4 h. The corrosion rate was calculated by normalizing the volume of hydrogen gas to the initial sample surface area and applying a numerical derivation. Then, the curves were smoothed using a moving average algorithm. An average of at least two independent measurements was calculated.

### 2.3. Surface Analyses

Before analyses, visual inspection of the surfaces was performed using a stereo microscope, Olympus. X-ray Photoelectron Spectroscopy (XPS) was used for the surface analyses of the samples. A Microlab 310 F VG-Scientific SEM/AES/XPS instrument ((VG Scientific Ltd., East Grinstead, UK)) was used. Images were recorded using Scanning Electron Microscopy (SEM). Due to the strong charging of the sample’s surface, the best-quality information was obtained by XPS, which provided the chemical composition of the surface and the chemical states of the individual elements, thus suggesting possible compounds.

For all of the XPS measurements, Al-Kα radiation at 1486.6 eV, with an anode voltage of 12.5 kV and an emission current of 16 mA (total power of 200 W), was used. The C 1s beneficial contamination peak at 284.7 eV was used for the energy-scale calibration. Pass energies of 20 and 100 eV were used for the survey and high-resolution measurements. The step size used in the high-resolution measurements was 200 meV, with up to 20 scans averaged in the measurement of one spectrum. The measurements and data acquisition were controlled with the Avantage 3.41v data-acquisition and processing software supplied by the SEM/AES/XPS equipment manufacturer. The commercially available Casa XPS software (Version 2.3.15, Casa Software Ltd., Teignmouth, UK) for XPS and AES data processing [23] was also used for further data processing, e.g., fitting the high-resolution peaks using components corresponding to the different chemical states of the elements. Reference XPS data were taken from [24,25].

#### 2.3.1. Experimental Parameters Specific to XPS Analyses before Immersion Testing

Before the XPS measurements, the sample surfaces were sputtered in cycles of 30 min (1800 s) for up to 90 min (5400 s) using an Ar^+^ ion beam of 3 keV with a 0.8-µA ion current over a 20 mm^2^ area, with a sputtering rate of approximately 0.15 nm/min. After each sputtering cycle, a quick XPS was taken, and when the C 1s stopped decreasing, the sputtering was stopped. With the parameters used, this corresponded to removing approximately 15 nm from the top of the sample.

#### 2.3.2. Experimental Parameters Specific to XPS Analyses after Immersion Testing

The XPS measurements were performed after sputtering with Ar^+^ ions in steps of 900 s for total sputtering times of up to 60 min (3600 s). An Ar^+^ beam of 3 keV was used at a 0.8-μA ion current over a square area of 16 mm^2^. At these parameters, the sputtering rate was approximately 0.2 nm/min, which means that after 60 min of sputtering, approximately 15 nm of material was removed from the surface.

## 3. Results

### 3.1. Analysis of Samples before Corrosion Testing

#### 3.1.1. Imaging

**Figure 1:**
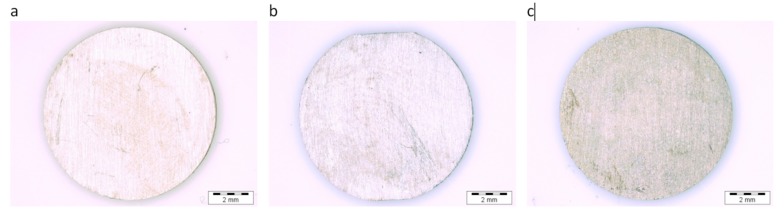
Stereo microscope images of representative samples X03 (**a**), Z5 (**b**) and ZX50 (**c**) before immersion in SBF.

The surfaces of the representative samples, provided for XPS analysis before immersion in SBF, can be seen in Figure 1.

#### 3.1.2. XPS of Samples before SBF Immersion

**Figure 2:**
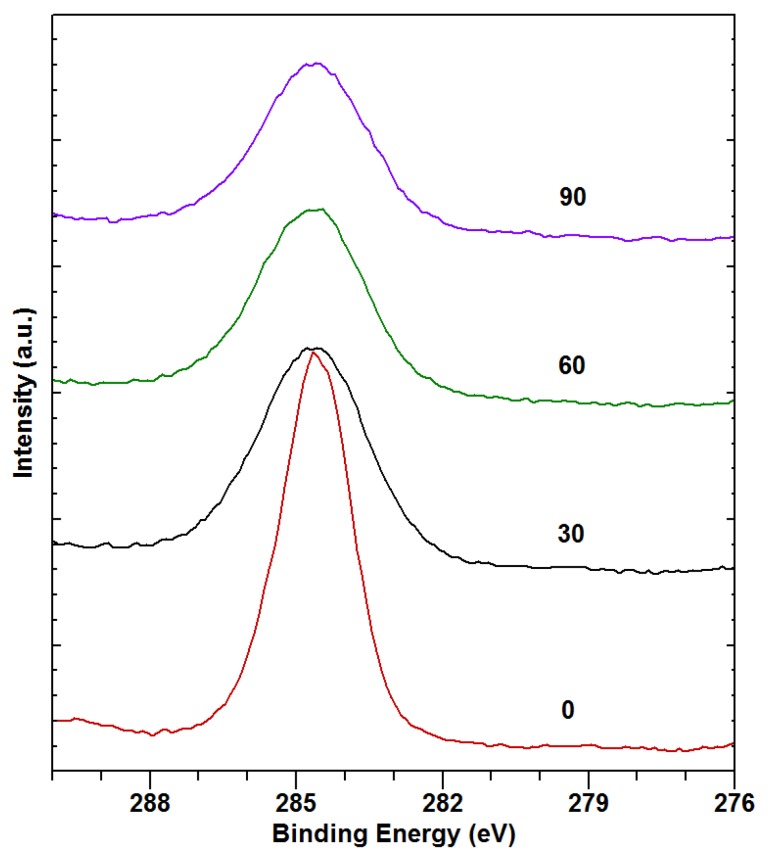
Typical C 1s peak intensities after different sputtering times (in minutes).

**Figure 3:**
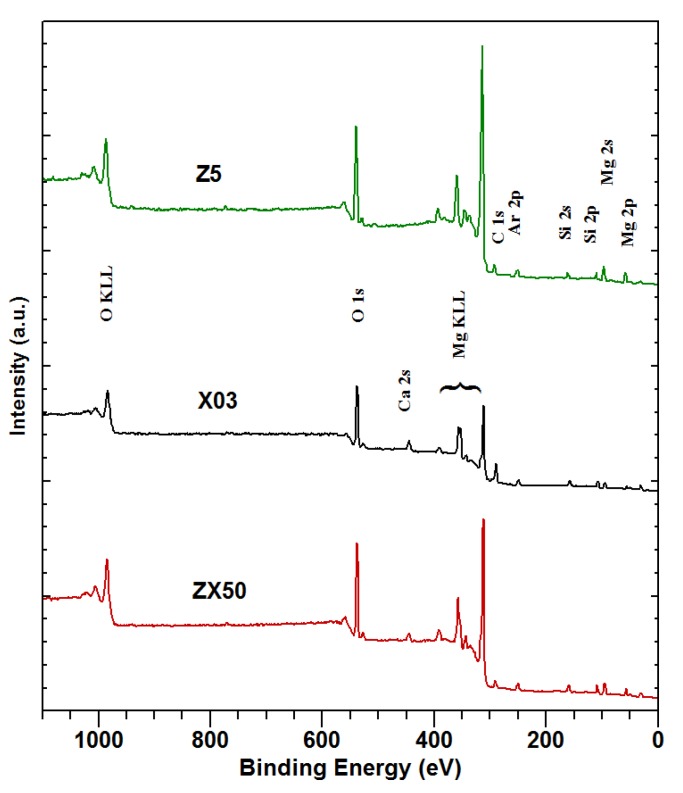
Survey X-ray photoelectron spectroscopy (XPS) spectra of sputtered samples ZX50 (Mg-0.3Ca-5Zn), X03 (Mg-0.3Ca) and Z5 (Mg-5Zn) before immersion in SBF.

The surfaces of two binary alloys, X03 and Z5, and one ternary alloy, ZX50, were investigated by XPS before the bio-corrosion immersion tests in the SBF. Figure 2 shows that apparently all of the sputter cleaning is already achieved after the first sputtering cycle. Full XPS spectra after the final sputtering cycle are shown in Figure 3.

Comparing the spectra of the sputtered samples (Figure 3), the following is observed: Mg appears on the surface of all of the samples. The binding energies are consistent with Mg oxides, phosphates and carbonates. Low-intensity Si 2p and Si 2s peaks are also observed. They most probably stem from contamination by silicon oil during the preparation of the sample. This is supported by the fact that no Si was detected in the initial composition of the samples. As expected, Ca is not observed in Z5. In X03 and ZX50, the binding energies are consistent with Ca oxides, carbonates, phosphates and silicates. Several O components appear in all of the samples, with binding energies consistent with different oxides, carbonates and phosphates. No Zn is observed in any sample. The small concentration of Zn (only 1.9 at%, see Table 1) suggests that the acquisition time, even for the Zn 2p peaks (Zn 2p3/2 at 1023 eV and Zn 2p1/2 at 1046 eV, the most intense of all Zn transitions), was too short. On the other hand, Ca 2s peaks, which should be of roughly the same intensity, are observed. This suggests that the surface layer of the sample is significantly enriched with Ca compared to the bulk (Table 3). Additionally, partial Zn depletion of the surface layer is also possible. Since no phosphorus is detected, it can be concluded that the surface is predominantly covered with an oxide layer, since no carbonate peak is observed in C 1s (Figure 2).

Comparing Table 1 and Table 3, a drastic Ca enrichment in the surfaces of Ca-containing samples can be found. This could be due to Ca precipitation on the surface and/or the leaching of Mg and Zn during polishing. A similar effect has been observed by Liu et al., where the Ca concentration in precipitates was an order of magnitude higher than in the matrix [6].

### 3.2. Evolution of the Corrosion Reaction with Time

**Figure 4:**
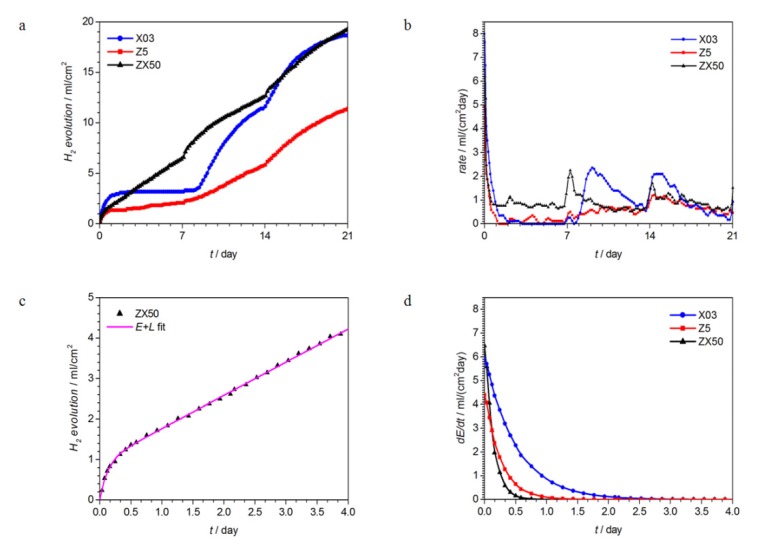
H_2_ evolution (**a**), the corrosion rates (**b**), H_2_ evolution fitted by *E* + *L* (**c**) and the rates for the exponential contribution (**d**).

In Figure 4a, the evolution of hydrogen during the immersion of samples in SBF is shown. The kinetics appears to be exponential at the very start of corrosion, before changing to linear or apparently constant (in the case of X03). The reaction boosts after the replenishments of SBF at 7 and 14 days can be clearly seen. After the replenishments, the kinetics seems to become more complex, especially in the Z5 sample. The rates of corrosion derived from the hydrogen evolution are shown in Figure 4b. The corrosion rates for Mg may differ depending on various parameters: the degree of its purity, heat treatment of the material, the type of electrolyte, the time of immersion, the crystal orientation, etc. [6,7,17,26,27,28,29,30,31]. As reported, Mg in the high-purity form has the lowest corrosion rate, below 1 mlcm^−2^day^−1^. However, heat treatment causes its corrosion rate to increase to 1–5 mlcm^−2^day^−1^ [30]. On the other hand, corrosion rates of as-cast pure Mg (melted from 99.98 wt% Mg), under a short time of immersion (i.e., 2 h) may be up to 17 mlcm^−2^day^−1^ [31], and in commercially pure Mg, exposed in SBF for 24 h, they are approximately 6–7 mlcm^−2^day^−1^ [26]. However, due to the fact that the degradation behavior of the Mg alloy is always related to its surrounding environment [31], and the chosen experimental set-up, the evolution of corrosion rates over time is rather complex (Figure 4b). A significant non-zero rate after first 2–3 days is only observed in ZX50. In Figure 4a, both the exponential start as well as the linear continuation of hydrogen evolution can be very clearly observed for ZX50. Thus, we tried to fit these first 4 days using the sum of an exponential and linear function, *E* + *L*. *E* = *a*(1 − e*^−bt^*) is the so-called increasing form exponential decay, which increases rapidly at first, and then levels off to some asymptotic value. It is used to describe the early stages of the reaction. *L* = *ct* + *d* is the linear component, supposed to correspond to the later stages of the reaction. A nearly-identical shape is found in the literature for H_2_ evolution tests on pure Mg using SBF with a TRIS/HCl buffer [26]. The results for ZX50 are shown in Figure 4c. The same fits were made for X03 and Z5, and the rates, *d*(*E* + *L*)/*dt*, were then calculated. The rates for the exponential part, *dE*/*dt*, are shown in Figure 4d. All of the *dE*/*dt* rates become zero after 2.5 days. The linear parts, *dL*/*dt*, for X03, Z5 and ZX50 amount to 0.06, 0.11 and 0.82 mlcm^−2^day^−1^, respectively. Thus, even if the linear part rates for X03 and Z5 are considerably smaller than for ZX50, they are not zero, as is suggested by Figure 4b. *E* and *L* could correspond to first-order and zero-order reactions taking place during the corrosion process. In the future, this could help in identifying the reactions involved.

### 3.3. Analysis of Samples after SBF Immersion

#### 3.3.1. Imaging

**Figure 5:**
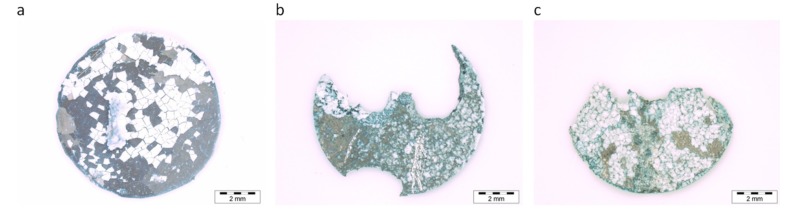
Stereo microscope images of samples X03 (**a**), Z5 (**b**) and ZX50 (**c**) after immersion in SBF.

Stereo microscope images of the three representative samples aimed at XPS analysis, after immersion in SBF, are shown in Figure 5. Comparison of the samples after three weeks of immersion in SBF (Figure 5) reveals the features and characteristics of exposed surfaces; parts of the samples are already missing in the case of both Zn-containing alloys, while the X03 alloy shows a rather homogenous corrosion behavior.

**Figure 6:**
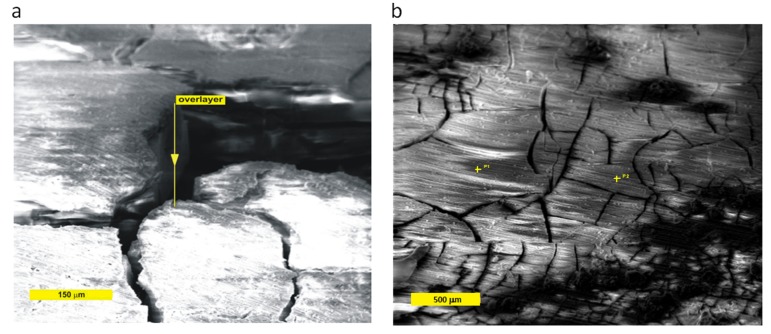
Typical low-magnification SEM images of cracks in the scaled degradation layer in sample X03 at higher (**a**) and Z5 at lower (**b**) magnification after immersion in the SBF.

SEM imaging of the surface was also attempted but, due to the strong charging of the surface, only two poor-quality SEM images were obtained, with brightness over-saturation on the upper surfaces (Figure 6). Nevertheless, at 60° tilt of the sample, the lateral surface of a grain in the crack could be observed in one of the SEM images (Figure 6a), and a rough estimate of the degradation layer’s thickness could be made, of the order of 100 µm. A somewhat larger area of the sample Z5 is shown in Figure 6b. Two points where localized Auger Electron Spectroscopy (AES) was attempted have been marked. However, no useful spectra were obtained due to strong surface charging. A tentative assessment of the surface layer thickness of the order of 100 µm could be made from the deep crack in the bottom part of Figure 6b. The dimensions of the scales on the other samples are somewhat smaller, but are roughly of the same order of magnitude; thus, it can be tentatively concluded that thicknesses of the degradation layers are 10–100 µm. The surface morphology is typical of Mg alloys exposed to buffered SBF, e.g., [26,27].

#### 3.3.2. XPS of Samples after SBF Immersion

**Figure 7:**
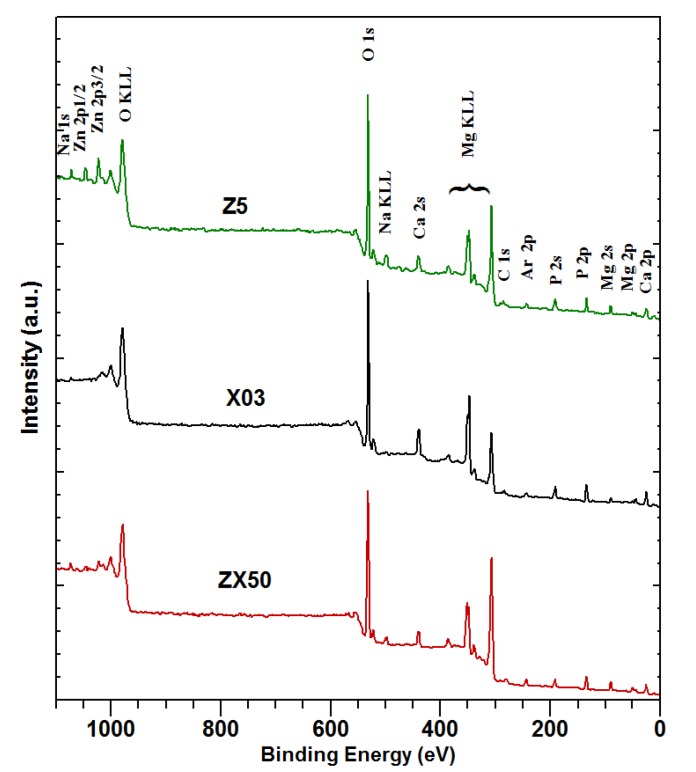
The survey XPS spectra of sputtered samples of ZX50, X03 and Z5 alloys after immersion in the SBF.

**Figure 8:**
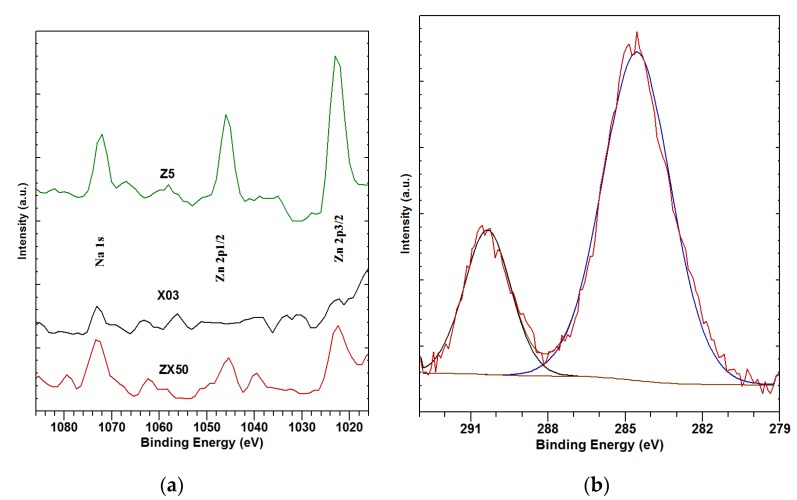
Narrow acquisition range XPS scans of 1016–1086 eV of the ZX50, X03 and Z5 alloys (from bottom to top) after immersion in SBF (**a**), and the HR XPS spectrum of C 1s fitted with components around 285 eV and 290 eV (**b**).

As concerns the investigations by XPS, several levels with increasing sputtering times of survey and XPS spectra were measured to ensure that the surface contaminants were removed by etching (Figure 7). The high-resolution spectra of some relevant elements were also measured (Figure 8).

Similar to the XPS spectra measured before the immersion in SBF, only the spectra from the finally-sputtered samples are shown in Figure 7, while the most of cleaning was already achieved after the first sputtering cycle.

In Figure 8a, a narrow acquisition range of XPS scans 1016–1086 eV for the ZX50, X03 and Z5 alloys (from bottom to top) is shown. Zn 2p3/2 at 1023 eV, Zn 2p1/2 at 1046 eV and Na 1s at 1073 eV peaks are clearly visible in the spectra corresponding to ZX50 and Z5, but not in those representing the X03 alloy. Since the measured Zn concentrations in the Zn-containing samples are in the order of 1 at% (Table 4), and since corresponding peaks can be clearly seen in Figure 8a, this confirms the suspicion of surface Zn depletion in the samples before SBF immersion. The positions of the Zn and Na peaks suggest chemical states corresponding to zinc oxide and sodium (hydrogen) phosphate.

In Figure 8b, the peaks corresponding to C 1s are shown. The larger component around 285 eV corresponds to the carbohydrate-based surface contamination, while the smaller component around 290 eV corresponds to C in the carbonates. The first component decreases and the second one increases with sputtering time. Most of the change, however, is already accomplished after the first sputtering cycle, as was the case with the carbon-contamination removal in the samples before the SBF immersion. This finding is observed in all of the samples.

From the measured spectra of the sputtered samples, elemental concentrations were obtained for all samples (Table 4). Only the carbon pertaining to carbonates is taken into account in Table 4. The share of this carbon is obtained by fitting the C 1s peaks with the components at 285 eV and 290 eV, as shown in Figure 8b. However, the concentration values in Table 4 suggest that the carbonates do not form an important share of the degradation layer.

Importantly, a considerable amount of Ca is detected in the degradation layer on the Z5 alloy (Figure 7, Table 4) in spite of none being present in the original alloy (as verified by the composition measurement, Table 1, and XPS measurement, Figure 3). This is most likely due to Ca^+^ and HCO_3_^−^ ions from the SBF forming calcium carbonate that is deposited onto the sample surface. Also, comparing Table 1 with Table 3, it is clear that before the SBF immersion, the surface concentration of the initially-low Ca concentration is increased in the Ca-containing samples, although Ca is not detected in the Z5 sample. This increase could be due to the non-homogeneity of the topmost layer of the sample. There is a significant further increase of the Ca concentration during the SBF immersion (Table 3 and Table 4). Since there is a significant Ca concentration on the Z5 sample after the SBF immersion, this Ca must have come from the solution. It is possible that some of the Ca on the X03 and ZX50 samples is also deposited from the SBF during immersion. It has also been found that during the phosphate deposition, the Ca/Mg ratio increases for approximately 10 h, which is followed by a slow decrease after >100 h of immersion [28].

Table 4 shows that the concentrations of Ca, P, Mg and O are significantly larger than the concentrations of other detected elements in the degradation layer. A simplifying assumption was that most of the overlayer consists of apatite, magnesium phosphate and magnesium hydroxide, with *p*, *q* and *r*, *p* + *q* + *r* = 1 as their respective shares: *p*[Ca_10_(PO_4_)_6_(OH)_2_] + *q*[Mg_γ_H_n_(PO_4_)_2_∙*x*H_2_O] + *r*[Mg(OH)_2_]; *n* is 1 or 2, depending on the phosphate type. The second term is a generic form of (mono, di, tri) magnesium phosphate hydrate, while the third term is magnesium hydroxide, which is an expected product of the Mg alloy corrosion in SBF [29]. Magnesium phosphate is indicated by the ratio of the calcium concentration to the phosphorus concentration, being significantly lower than 1.66, which corresponds to the apatite (Table 4). The concentration data from Table 4 were used to calculate *p*, *q* and *r* for different values of *γ*, i.e., different types of magnesium phosphate (see Table 5). Determining *x* was not attempted since it is difficult to estimate the share of oxygen as a contaminant. From Table 5, it is clear that apatite appears in all of the samples for all *γ* values, usually with a low share of around 0.1. Magnesium phosphate hydrate appears with shares comparable to those of magnesium hydroxide, while in two cases, it is found to be even larger. The significantly larger share of magnesium hydroxide compared to magnesium phosphate hydrate in sample Z5 is consistent with a low phosphorus vs. magnesium concentration compared to that of the other samples. The apparently non-existent magnesium hydroxide in X03 for *γ* = 3 suggests that tri-magnesium phosphate hydrate does not form in that sample.

## 4. Discussion

From Table 5, it can be seen that in X03, the share of Ca and Mg phosphates is higher than in Z5 and ZX50, while the share of Mg(OH)_2_ is lower. This may again point to in-depth hydr(oxide) formation that degrades the sample (Figure 5), as suggested in the discussion of the reaction dynamics (3.2.2.).

A comparison of the obtained results with the literature is limited in this work, since corrosion rates depend strongly on the measurement technique, corrosion medium and pH buffer capacity [32,33]. These were not varied and assessed in the present study. However, when comparing the three tested alloys, it can be seen from Figure 4b that the corrosion rate is highest for the ternary ZX50 alloy—especially in the first days of immersion—while the binary Z5 alloy shows the lowest total hydrogen evolution (Figure 4a). These findings are consistent with our earlier report, where shorter times and an alternative technique for investigating the corrosion process were used [34].

When considering a material for biodegradable implant applications, at least two aspects of the degradation behavior are important.

First, the overall degradation rate should not be so high that the hydrogen gas evolving during the corrosion reaction leads to the formation of bubbles in the surrounding tissue [35]. This points to the X03 alloy as most appropriate of the three.

Second, the corrosion behavior should be homogeneous, and no pitting or localization of the corrosion reaction should occur, as this may lead to premature failure of the implant. An optical inspection of the samples after three weeks of exposure to SBF (Figure 5) shows that in case of both Zn-containing alloys, parts of the samples were already missing, while the X03 alloy showed rather homogenous corrosion behavior. Thus, this alloy may be the best one to be considered for implant applications—at least from the viewpoint of degradation behavior. However, the mechanical properties of this alloy are usually insufficient for appropriate structural performance, and therefore, other biocompatible alloying elements, e.g., Zn, should be added for solid-solution or precipitation strengthening [7,20].

The mechanical properties, mainly the strength, are also crucial in view of the applicability of biodegradable implants [5,7,16,17,20]. It should be noted, therefore, that all of the alloys investigated in this study were in solid solution-treated condition. Different processing techniques can significantly affect not only mechanical properties, as reported by Horky et al. [36], but also the corrosion rate of Mg-alloys, as shown by Ojdanic et al. [37,38]. The low hydrogen evolution rate in the alloy Z5 and corrosion rate in alloy X03, along with the high corrosion rate in ZX50, indirectly suggest that the heat treatment of the last was not optimal. This may lead to residual primary precipitate particles remaining in the microstructure, which in turn leads to an accelerated corrosion rate. These will be further investigated and optimized in forthcoming studies.

Large missing parts in the Z5 and ZX50 samples (Figure 5b,c) may also suggest that *E* and *L*, the exponential and linear reaction components, are responsible for the surface degradation layer growth and in-depth (hydr)oxide growth, respectively. The reaction in sample X03 virtually stops after 2 days (Figure 4a,b) when the exponential component *E* approaches 0 (Figure 4d). It can be speculated that during this phase, more surface oxide forms than in samples Z5 and ZX50, where *E* disappears faster (Figure 4d). This surface oxide on X03 serves as a barrier against in-depth corrosion.

## 5. Summary and Conclusions

Two binary biodegradable Mg-alloys and one ternary biodegradable Mg-alloy (Mg-0.3Ca, Mg-5Zn and Mg-5Zn-0.3Ca, all in wt%), X03, Z5 and ZX50, respectively, in solid-solution state were investigated before and after in vitro bio-corrosion immersion tests in SBF.

The surface chemical composition of all the samples before the immersion in SBF can be described as follows: several oxygen components were observed for all of the samples, with the O 1s binding energy being consistent with different hydroxides, oxides, phosphates and carbonates. Mg 2s and Ca 2s binding energies are consistent with magnesium (hydr)oxides and carbonates. The carbonates were only present in small concentrations or not at all, since a corresponding carbonate component of the C 1s peak could not be observed. Before immersion, Zn was not observed at all on the surface of any of the sample. This might be due to the low average concentration of Zn (at the XPS detection threshold), possibly enhanced by a non-homogeneous composition of the surface layer relative to the bulk. At the same time, significant Ca enrichment in both Ca-containing samples was observed.

The progress of reactions during the SBF immersion was followed by monitoring the evolution of H_2_. An exponential–linear model was proposed for the reaction kinetics during the first stage of immersion.

The XPS results suggest that the typical bio-corrosion products on the Mg-0.3Ca, Mg-5Zn and Mg-5Zn-0.3Ca alloys consist of Mg, Ca, P and O. The surface concentrations are consistent with magnesium hydrogen phosphate and magnesium hydroxide, with up to 20% of apatite (calcium phosphate hydroxide).

Based on the reaction dynamics and the post immersion surface composition results, as well as optical images of the post immersion samples, it is suggested that the linear contribution to the reaction rate is related to the in-depth corrosion and sample degradation.

Considering the applicability of the analyzed alloys as materials for biodegradable implant applications, the overall degradation rate points to the Mg-Ca alloy (X03) as the most appropriate of the three. As for the structural integrity of the sample after long immersion periods, the optical inspection of the samples again points at this alloy.

Nevertheless, the considerations of higher structural properties, when required, suggest also the alloy ZX50 as a potent candidate upon further optimization of the thermo-mechanical treatment.

## Figures and Tables

**Table 1:** The chemical compositions of the alloys in wt% and at%, as measured by X-ray Fluorescence Spectroscopy (XFS) before immersion into simulated body fluid (SBF).

Nominal Composition	Mg-0.3Ca		Mg-5Zn		Mg-5Zn-0.3Ca	
Naming Convention	X03		Z5		ZX50	
**Measured Composition**	wt%	at%	wt%	at%	wt%	at%
**Ca**	0.3	0.2	−	−	0.3	0.2
**Zn**	−	−	5.0	1.9	5.0	1.9
**Mg**	99.7	99.8	95.0	98.1	94.7	97.9

**Table 2:** The composition of SBF (ionic concentrations in mmol/L) [1].

Ion	Na^+^	K^+^	Mg^2+^	Ca^2+^	Cl^−^	HCO_3_^−^	HPO_4_^2−^	SO_4_^2−^
**Concentration**	142.0	5.0	1.0	2.5	109.0	27.0	1.0	0.5

**Table 3:** The binding energies (BE) and atomic concentrations in the measured samples. Different components of O correspond to different compounds of O.

Sample	X03		Z5		ZX50	
XPS Peak	BE/eV	c/at%	BE/eV	c/at%	BE/eV	c/at%
**Ca 2s**	440.3	12.9	−	−	439.4	6.0
**Mg 2s**	90.4	25.7	89.7	40.7	89.8	36.0
**O 1s 1**	532.4	53.1	531.9	52.6	532.0	48.4
**O 1s 2**	529.9	1.0	530.5	3.0	531.5	2.8
**O 1s 3**	533.5	6.4	533.6	5.9	533.5	6.8

**Table 4:** Elemental concentrations in at% for elements detected at the top of the degradation layer in all measured alloys.

Sample	X03	Z5	ZX50
c(Na)	0.4	0.7	1.5
c(Zn)	0.0	1.0	0.5
c(O)	59.1	56.6	58.9
c(Ca)	14.5	8.9	8.9
c(C)	2.0	3.1	2.8
c(P)	15.2	11.0	13.2
c(Mg)	9.2	19.0	14.3

**Table 5:** Parameters *p*, *q* and *r* of the proposed apatite–phosphate–hydroxide model for different types of phosphate.

Sample	X03			Z5			ZX50		
*γ*	1	2	3	1	2	3	1	2	3
***p***	0.15	0.20	0.30	0.05	0.05	0.20	0.05	0.10	0.10
***q***	0.30	0.35	0.70	0.15	0.15	0.05	0.25	0.35	0.55
***r***	0.55	0.45	0.00	0.80	0.80	0.75	0.70	0.55	0.35
